# Understanding emerging patterns and dynamics through the lenses of the cyber-physical universe

**DOI:** 10.1016/j.patter.2022.100601

**Published:** 2022-11-11

**Authors:** Mauro Lombardi, Simone Vannuccini

**Affiliations:** 1 University of Florence, Florence, Italy; 2 Science Policy Research Unit (SPRU), University of Sussex, Brighton, UK

**Keywords:** cyber-physical universe, ubiquitous computing, information technology, artificial intelligence, decision making

## Abstract

The complex interaction among contemporary techno- and socio-economic processes has set the stage for the emergence of a cyber-physical universe, the novel landscape in which agents behave and interact, and which is centered on the fundamental role played by information and computation at all levels. In this paper, we weave into a single analysis the different threads that lead to (and characterize) the cyber-physical universe and outline a map of its building blocks and the complex dynamics at work in the new environment. The resulting description is used to assess how decision-making processes should evolve in order to be able to address the opportunities and challenges of the current era of deep and extended changes. The analysis offers an encompassing interpretative grid to understand and unpack patterns in the contemporary socio-technical systems that experience a fundamental informational turn; this can inform new research trajectories and help open up new areas for scientific inquiry.

## The bigger picture

This paper proposes a new organizing concept—the cyber-physical universe—to describe the current overarching socio-technical-economic landscape. The cyber-physical universe is the result of information technology permeating every aspect of reality and blurring the boundaries between the physical and digital domains, at different scales, a process that changed in a fundamental manner the logic and rules of evolution of contemporary socio-economic and techno-scientific systems. By outlining the steps that lead historically to the cyber-physical universe, as well as its properties and the dynamics it enables or influences, we are able to shed a light on the higher-level “machinery” within which humans act and science (including data science) develops. In particular, we trace the path of decision-making augmentation culminating in the use of artificial intelligence technologies. As a map of the current landscape, we posit that the cyber-physical universe can help direct scientific exploration, production strategies, and decision making more generally. The analysis we conduct is useful as it offers an encompassing interpretative grid and basic principles to understand and unpack patterns in systems that experience a fundamental informational turn; this can inform new research trajectories and help open up new areas for scientific inquiry.

## Teaser

We present a new organizing concept—the cyber-physical universe—that places into perspective the complexity and interdependence of current socio-techno-economic transformations, from the global to the nano scale and at the intersection of physical and digital realities. As a map of the current landscape, the cyber-physical universe can help direct scientific exploration, production strategies, and decision making more generally. We show how this scheme is useful to creating connections and order among the trends and patterns uncovered by different disciplines.

## Introduction

We live in a time of great changes. The transformations triggered by the coronavirus disease 2019 (COVID-19) pandemic add to and amplify three socio-technical and techno-economic processes, which, in turn, are at the basis of three joint crises: the biological-environmental (climate change), the techno-productive, and the economic-financial. The entire world seems on a path approaching what scholars in different disciplines consider situations at high risk of “unwanted collapse.”^1^ An unwanted collapse is a tipping point, or a catastrophic bifurcation point, “where a minor trigger can invoke a self-propagating shift to a contrasting state.”^1^

The claim that the world (or a share of it) is on the verge of a collapse is recurring, and often appears at relevant historical nodes; for example, in phases of transition between established and upcoming techno-economic paradigms.^2^ To put it with Gramsci, “the crisis consists precisely in the fact that the old is dying and the new cannot be born; in this interregnum a great variety of morbid symptoms appear.”^3^ This explains the cyclicality (and, thus, the recurring fads) of the theories of cycles in social sciences, such as that of regulation, of world systems, or of long waves.^4^ However, not all catastrophes—in the sense of catastrophe theory—are alike. In this paper, we make the case for the uniqueness of the current unwanted collapse to come, and discuss how decision making must start accounting for it in a structural manner. In fact, the three joint crises behind the potential global tipping point occur after decades of evolutionary acceleration induced by an array of factors and forces that have shaped a “hyper-connected” world. This hyper-connected world is characterized by the emergence of a multiplex of self-organized local and global interaction structures and processes within and between different domains.

Our analysis outlines a map of the deep and extended changes that are currently unfolding and shaping the hyper-connected world we live in. This map is described by a collection of fundamental coordinates: building blocks, concepts, and principles that we single out while we also highlight their interconnections. The organizing notion that connects the dots is the cyber-physical universe, an informational and physical landscape drawing the boundaries for actions and transformations to unfold. The cyber-physical universe is the result of information technology permeating every aspect of reality and blurring the boundaries between the physical and digital domains, at different scales.

The concept of cyber-physical universe can help direct scientific exploration, production strategies, and decision making more generally. What we propose is an exercise in pattern spotting, from the global to the nano-level of analysis, and back; our analysis provide a lenses to interpret emerging dynamics and patterns in socio-techno-economic systems as they experience a fundamental computational and information turn. By outlining the steps that lead historically to the cyber-physical universe, as well as its properties and the dynamics it enables or influences, we are able to shed a light on the higher-level “machinery” within which humans act and science (including data science) develops. In particular, we trace the path of decision-making augmentation culminating in the use of artificial intelligence [AI] technologies. The key take-home message of the paper is that, by “giving a language” to machines through a progressive codification of information in digital form and a technology-enabled computational turn in all domains of human activity, humans have changed the basic rules of evolution of socio-economic and techno-scientific systems.

We show how our theoretical scheme is useful to creating connections and order among the trends uncovered by different disciplines. In particular, we suggest that a shift to “adaptive strategic thinking” is required for actors to survive and succeed in the cyber-physical universe. The novelty of our contribution lies in the unique combination of several literature strands, which we use to stress the convergence of different and often non-proximate scientific domains around similar perspectives and problems. The snapshot analysis we propose is necessarily systemic, complex, non-linear, and recursive, as it mirrors the nature of the landscape we study.

## Coordinates for the current era: The cyber-physical universe

As a preliminary to our work, we outline a set of (improperly speaking) axioms on the nature of socio-techno-economic systems. These basic statements, compiled from contribution in innovation, technological change, and complexity studies, define the paradigm of analysis from which our insights will descend. They are (1) the adoption of a general definition of technology as “any intentional extension of a natural process, that is, of processing of matter, energy, and information that characterize all living systems”^5^; (2) the proposition that “a society cannot develop unless an adequate infrastructure for the movement and processing of matter, energy, and information already exists”^5^; (3) material and immaterial infrastructures are evolving multi-layered networks of structured knowledge^6^^,^^7^; (4) the evolution of complex adaptive systems have wave-like properties^8^; (5) sequences of socio-technical landscapes are complex and interrelated processes evolving toward asymptotic stationary equilibria, while they are every now and then interrupted by distributed discontinuities^9^; (6) deep and extended changes occur when founding rules are changed, and their effects propagate even after a long time.

In the section “introduction,” we mentioned the three distributed discontinuities (crises) that are affecting and transforming—potentially in an abrupt, unwanted-collapse manner—the socio-technical landscape, a change in line with the pattern summarized by point (5) above. In this section, we outline the contours of the new landscape in the making and the dynamic forces shaping it. Our idea is that ongoing complex developments, approximated by changes in (3) and following the path captured by (4) and (5), are producing (6). We describe these complex developments below.

The point of departure for our analysis is the following: the exponential increase of computational power and storage capacity, the pervasiveness of information-processing devices (ubiquitous computing and connectivity), advances in digital technologies (namely those enabling digitalization, or the translation of analog signals into machine-readable formats), and the creation of software systems (including AI algorithms) able to process an increasing amount of information flows from all over the world, have been key drivers of a generative process of intermingling networks at multiple scale and across traditionally different socio-economic activities. This generative process has definitively decoupled the locus of (economic) value generation and that of information production and processing, which now is ubiquitously distributed. Thanks to the consequent triggering of cross-scale positive feedback among these interdependent dynamics, thereby feeding systemic complexity at both the local and global level, a new global landscape has emerged.

This new global landscape is characterized by the following properties. (1) The development of self-organizing processes, able to manage hyper-scale infrastructures, which have been essential drivers of the formation of hyper-structures.^6^^,^^7^ (2) Techno-scientific advancements, especially in (but not limited to) digital technology, allow representing (codifying) real processes and outputs from the nanoscale to the ordinary and global scale. Each codified “object” in the natural world becomes a source of fine-grained, real-time digital data and can be paired with its own digital representation, which can be labeled digital model, digital shadow, or digital twin, depending on the flows of information (one way, bidirectional) between the environment, the physical object and its representation.^10^ This kind of theoretically complete 1:1 map from the sub-atomic world to whatever level deemed appropriate for designing processes and outputs can lead, in essence, to perpetual self-production, creating what Zittrain^11^ has called a “generative space” of ideas and knowledge. (3) The closed world of Newtonian theory, to paraphrase Koyré,^12^ is unfit to explain the new landscape. Humans now live in an open-ended universe,^13^^,^^14^ which is continuously expanding and evolving. (4) As a result, we also experience an accelerated expansion of the digital universe, parallel and tightly linked to real processes and their dynamics. We define this complex and dynamic intermingling the cyber-physical universe, within which real and digital processes interact and influence each other to the point that sometimes it becomes impossible to distinguish real from virtual. (5) The openness of the cyber-physical universe implies that the Newtonian mechanistic clockwise “in which big problems can be broken down into smaller ones, analyzed, and solved by rational deduction”^15^ is no longer working. The machine metaphor is outdated and inappropriate to understand what is happening within the “Earth System,”^16^^,^^17^ where the standard model based on linear cause-effect relations does not work. The globalization of processes, within which goal-oriented interactors (individuals, collective entities) pursue their goal(s), give rise to interlocking relationships, with relational topologies emerging from exploratory activities performed in different techno-scientific search spaces. The cyber-physical universe is the world of non-linearities, because the agents populating it evolve on the basis of exchanging information, constructing and modifying systems of beliefs, cognitive procedures, mental models and system of rules, all endogenously shaped by the topology and nature of multi-level and multi-domains interactions. These non-linear and systemic dynamics of cross-influences has triggered an exponential acceleration of change for the whole Earth system.

In summary, profound techno-economic transformations and their co-evolution extended to the whole Earth system are producing a novel, unprecedented landscape within which actors operate: the cyber-physical universe. This landscape is characterized by multi-level complexity, non-linearities, and restless endogenous reconfiguration, and is made coherent by the pervasiveness of its informational nature, so much that every phenomenon can be read through the lenses of information.^18^ Given that, and paraphrasing David Deutsch,^19^ we advance the following statement: The entire planet has become a techno-social system, where information technologies constitute the “fabric of reality.”

Such an unprecedented configuration of reality shapes the set of opportunities and challenges actors face. At this point in the discussion, three issues deserve to be addressed, which correspond to the sections of this paper: (1) what made the advances in techno-science that molded the cyber-physical universe possible? (2) What in particular has changed in the landscape around us? (3) On which mental models (paradigms) should decision-making processes be based, now that we are immersed into the cyber-physical universe?

## The trajectory (over centuries) toward the cyber-physical universe

If the cyber-physical universe, with its blend of informational and physical nature, is the novel global context for all actors, a first question to address is how we got there or, in other words, which forces and dynamics contributed to its formation over the long run. In this section, we outline three fundamental steps in the evolution of human attempts to represent the world that, cumulatively, have set the stage for the cyber-physical universe to emerge.

The first step is the discovery that the written language of the world can be binary. Philosophers have always questioned the nature of mathematics and geometry, as well as their relationship. A watershed event was the publication of Galileo’s The Assayer, where the scientist posits that the universe is an all-encompassing book written in mathematical language. For centuries before Galileo’s claims, humans have attempted to represent the world through a numeral system. The diffusion of the decimal number system (also called Indo-Arabic), which had numerous advantages compared with the Roman numeral-based system, has not stopped the search for different non-decimal numeration, such as binary and duodecimal, as documented by Glaser,^20^ as these were a potential source of utilitarian benefits. A big leap took place in the seventeenth century thanks to Leibniz, who for the first time in history elaborated the set of numbers from 0 to 15 in binary terms (see Leibniz’s letter to the Duke of Brunswick, 1697, reprinted in Glaser^20^). The importance of the binary representation by Leibniz should not be underestimated: the possibility to represent everything through 0s and 1s, even if conceived for theological reasons, has opened an enormous space for the development of human knowledge.

The second step is that insurmountable limits of human reasoning open up an unthinkable space of potentialities. About two and half centuries after Leibniz, Kurt Gödel^21^ wrote an article in which he demonstrated the undecidability of propositions belonging to a logical-formal system such as that of the Principia Mathematica by Whitehead and Russell. The achievement, known also as the First Incompleteness theorem, is above all remarkable from the point of view of the philosophy of logic, but at the same time it displays a crucial feature: it has strong similarities with a modern computer program.^22^ Many years before the invention of electronic calculators, Gödel was designing a logical procedure through which to formalize the “same issues that those designing programming languages and those writing programs in those languages would be facing.”^22^ In brief, Gödel introduced an algorithmic approach as a method of proof. The possibility to formalize the reasoning process in such a way that it is possible to demonstrate in a definitive manner even the impossibility of axiomatizing within logical-formal systems, can be likened to the “invention of a method of inventing”^23^: new knowledge is generated by the parsing of existing knowledge through operations that describe a set of rules of transformation.

The third step is the final leap to “abstractization” of human reasoning, subsequently embedding it into a real machine. This step was accomplished thanks to the contributions of two of the most important personalities in the field of the theory of computation. The first is Alan Turing, who in 1931 analyzed the computation process based on a further mathematical abstraction represented by an a-Machine, commonly known as a Turing machine. His result: anything computable by an algorithm can be computed by a Turing machine.^22^ The completion of the third step took place thanks to John von Neumann, who in 1930 had already understood the revolutionary content of Alan Turing’s speech in front of the most eminent scholars of the twentieth century, gathered in Könisberg, where he anticipated the ideas expressed in the 1931 article. von Neumann had already autonomously reached Gödel’s conclusions regarding the problem of the undecidability of propositions in the context of logical-formal systems. However, once the results obtained by Gödel were known, von Neumann no longer dealt with logic and devoted himself to the development of powerful computation machinery. In fact, von Neumann elaborated the famous Draft Report on the Electronic Discrete Variable Automatic Computer (EDVAC) computer (1945), in which essentially he proposed a device modeled on the “universal Turing machine.” With that, the von Neumann architecture was born, which is the embedding into hardware of the sequential (Turing) model of computation, still the prevalent architecture on which today’s computers are based.^24^

At the end of these three major steps, Leibniz’s dream of creating a “*characteristica universalis*,” that is, a symbolic system capable of representing human thought, and all the fundamental concepts and real processes using the binary system, beyond the syntactic differences existing between the different languages, seems to have come true. In reality, the developments we described set us on a path leading well beyond Leibniz’s dream. The binary system and the von Neumann architecture led the way to information technologies, which have been enhanced in the last few decades to the point of becoming what we have called the fabric of reality, a fundamental infrastructure, which in turn interacts and is in a superposition with physical processes to form a global whole: the cyber-physical universe. In a sense, the idea of the cyber-physical universe is closely related to the notion of planetary-scale computation, organized around the model of “the stack.”^25^ However, rather than discussing the political economy implications of the fabric of reality (even though we will touch upon that in section “a paradigm shift for decision making”), we take a techno-economic perspective to dissect the novel landscape: in the cyber-physical universe, countless sources of information and novelties are continuously generating unexpected impulses: individual and societal demands widespread at the international level; need for strategic resources, such as food, energy, water, or Rare Earth Elements^26^; techno-scientific advances; as well as competitive pressures between companies and countries.

In line with what we mentioned in the section “introduction,” the three steps outlined here represent an excerpt of the journey through which humans “gave a language” to machines. This is an unprecedented innovation, as well as what makes the current transformations unique, because it changes at the roots the basic rules driving the evolution of socio-techno-economic systems. The next step in our analysis is to “dissect” the new environment, by mapping its features and properties.

## What has changed in the landscape around us? Human decision-making processes facing a new complex environment

The analysis so far allowed us to frame a new, global, and extremely variable landscape, in which human decision processes must unfold. In this section, we describe the ingredients of the new landscape, as well as some of the changes induced upon the agents interacting with it. More precisely, we single out what is a new ontological space for actors, populated by cyber-physical systems; the evolution of humans’ external memory field (EMF) and its implications for information processes; the emergence of new tools for modeling the world; new properties of processes and products; and the move away from the traditional concept of firm toward innovation eco-systems.

### A new ontology of agents and cyber-physical systems

A first key feature of the cyber-physical universe is that it reshapes radically the “ontology of agents,” or ontological space.^27^ By ontology of agents we mean a conceptual space that agents themselves construct and define according to their ability to frame processes and events, representing the real world and the entities that populate it, and that can contain opportunities and challenges. The space of action of the agents depends, in fact, on their “*Umwelt* (subjective universe) [which] is governed, in all its parts, by the meaning it has for the subject …. [and] is altered and reshaped until it has become a useful meaning-carrier.”^28^ In the present era, the ontology of agents must be defined in relation to new components, in the light of the unfolding interlocking relationships among nested networks and processes at the global level. Furthermore, the concept can be applied to any “acting” entity, given the common informational fabric of the reality.

We claim that the crucial agents populating the new ontological space are cyber-physical systems (CPSs). CPSs “are integrations of computation with physical processes. Embedded computers and networks monitor and control the physical processes, usually with feedback loops where physical processes affect computations and vice versa.”^29^ Given the continuous expansion of the info-sphere^30^ and of what W. Brian Arthur calls “The Second Economy,”^31^ real activities unceasingly generate signals and information, giving rise in human minds to an ontological space teeming with multidirectional interconnections. As CPS “integrate physical dynamics and computational systems,”^32^ they become key actors in aiding, shaping, and steering human decision making.

### Humans and their EMF

While CPS are the crucial agents of the new ontological space, another fundamental component is worth examining: what Merlin Donald calls the EXMF.^33^ In Donald’s words, “the EXMF usually consists of a temporary array of visual symbols immediately available to the user. The symbols are durable and may be arranged and modified in various ways, to enable reflection and further visual processing.”^33^ In analyzing the evolution of the human mind and cognition, Donald distinguishes three transitions in the representational systems created by the brain during evolution. The adaptive emergence of the most recent one is the extension of “visuocognitive operations into, and becoming a part of, an *external symbolic system*”^33^ (italics added). Starting from the invention of written language, many graphic and visual tools have been created through interactions among people and more in general with the environment. This evolution has been the result of the attempt to bridge an ever-renewed gap between acquired knowledge and at the same time the need for new knowledge to solve problems. Indeed, the symbolic use of graphic devices has been enriched over the centuries through different forms of expression (artistic, technical, scientific, and so on). This unfolding has come about until the turning point (we add) of the binary system proposed by Leibniz, who provided an extremely powerful impetus to the development of an essential cognitive workspace, thanks to the introduction of a symbolic system inherently tending to represent the world in its entirety (universality), starting from basic principles and building on them rules and systems of rules. Hence, our discussion in section “the trajectory (over centuries) toward the cyber-physical universe” integrates with and broadens Donald’s original view.

### Information processing and generativity

Since CPS combine computation, communication, and physical dynamics, while the EXMF has become an expanding universe of both organized information flows and chaotic information particles, it is not surprising that human mental frames have been striving to pursue ever-greater computational power and ever more sophisticated representational systems. Indeed, cumulative feedback loops in which information-processing devices are used to produce new information (in turn feeding into the working of information-processing devices) have powered a sort of arms race between knowledge-accelerated growth and the tools to master it. As we discussed in section “the trajectory (over centuries) toward the cyber-physical universe,” the binary system was a key driver in feeding the continuous expansion of the info-sphere and consequently in making information technologies the “fabric of generativity,” as defined by Zittrain^11^: “Generativity is a system’s capacity to produce unanticipated change through unfiltered contributions from broad and varied audiences.” Generative systems occur when information flows, possibly coming from countless sources, self-organize based on the congruence between shared interests, values, paradigms, and worldviews, or “simply” because the agents share compatible research guidelines and objectives. The novelty of our age is that, thanks to information technologies, generative systems are the drivers and the result of global interconnections. In the cyber-physical universe, generative systems show particular features: (1) scalability, due to ubiquitous computing and connectivity; (2) adaptability, as physical architectures and software systems unceasingly evolve, in this way allowing more and more information to be created and/or processed; (3) progressive blurring of boundaries between material and immaterial processes, thanks to their integration enabled by the global spreading of CPS.

### New tools for modeling the world from the nanoscale to the ordinary scale and global level

The ingredients of the cyber-physical universe we outlined endow humans with tools to model the world with unprecedented precision. The current informational fabric of reality is grounded in the centuries of advances in knowledge representation we discussed in section “the trajectory (over centuries) toward the cyber-physical universe,” but there are other visions and basic techno-scientific discontinuities that inspired and kicked off the blurring of the digital and physical worlds. For example, in 1945, Vannevar Bush, Director of the Federal Office of Scientific Research and Development, envisions a “future device for individual use, which is a sort of mechanized private file and library. It needs a name, and to coin one at random, ‘memex’ will do. A memex is a device in which an individual stores all his books, records, and communications, and which is mechanized so that it may be consulted with exceeding speed and flexibility.”^34^ “Wholly new forms of encyclopedias will appear, ready-made with a mesh of associative trails running through them, ready to be dropped into the memex and there amplified.”^34^ This vision “has inspired a variety of other research endeavors, from information retrieval to distributed hypertext systems.”^35^ However, the memex was an analogical calculating machine and only in the early 1960s did Douglas Engelbart create the first electronic hypertext with the explicit aim of augmenting human capabilities. In his “augmentation framework,”^36^ Engelbart, explicitly referring to Bush, depicts a computer-assisted architect: “Let us consider an augmented architect at work. He sits at a working station that has a visual display screen some three feet on a side; this is his working surface, and is controlled by a computer (his ‘clerk’) with which he can communicate by means of a small keyboard and various other devices.” The vision of a computer as a clerk and the possibility of communicating through many other devices is the embryonic representation of the personal computer as a digital assistant, working with the architect in an Internet of processes. Another visionary technologist was Mark Weiser, computer scientist and chief technology officer (CTO) at Xerox PARC. There, he coined the expression “ubiquitous computing” and imagined the computer of the twenty-first century,” composed of many devices, such as hardware and software connected by wires, radio waves and infrared, and computer scratchpad.^37^^,^^38^ Finally, another milestone moment influencing the formation of the cyber-physical universe is Richard Feynman’s famous lecture “There’s Plenty of Room at the Bottom,” as on that occasion an incredible boost was given to the birth of nanosciences.

From the memex to the computer clerk and Weiser and Feynman’s visions, the embedding of informational language into technology has set humanity on a path of capability augmentation for discovery and creation. Further along this path, the continuous development of computational power and the information-processing loops we mentioned have opened completely new worlds (scales). It is now possible to computationally design one-dimensional, two-dimensional, and three-dimensional materials (including new semiconductor technology and nano-electromechanical systems [NEMSs]), and also four-dimensional (i.e., meta-) materials, which can be used in the production of products such as integrated circuits or nanorobots. Some of these materials do not exist in nature—as far as we know—and are created by engineering them at the atomic and sub-atomic level, and then building them up to the scale of everyday life, in what is labeled “multi-level materials design.”^39^^,^^40^^,^^41^^,^^42^ Basically, a computational and integrated modeling of the entire production process at every scale is currently underway, through what is called “integrated computational engineering” (ICME).^43^

In summary, the new tools enabled by the techno-scientific advances in the cyber-physical universe can help actors identify unexpressed potentials of materials, processes, and products, thus feeding the acceleration dynamics characterizing contemporary generative systems.

### New properties of processes and outputs

In the cyber-physical universe, each process or output (e.g., a product) tends to be the result of a diverse set of technologies, i.e., knowledge domains that are dynamically combined through intersections, overlaps, and convergences between different disciplinary fields. As in the present era, the identification of techno-productive problems and the search for their solutions occur within the new global landscape, and outputs assume a variable configuration, that is, a multi-technology and multi-disciplinary composition, as a mix of traditionally separate knowledge bases. More knowledge-intensive components are connected in such a way as to perform some functions, which can vary depending on the context in which they are inserted and on the degree of embedded intelligence in the algorithms that are involved in the process, which can, in turn, be transformed and adjusted depending on evolving human needs. Thus, products become smart, connected, and complex. Moreover, they acquire a new property: they can be rationally (purposefully) imagined and designed as sets of variable functionalities. As products derive from integrated physical and virtual-digital activities, their interacting with the cyber-physical universe, where multiple and repeated feedback between producers, consumers, technical-scientific domains, and socio-economic dynamics take place, feeds the emergence of new requirements, which can be matched through changing physical architecture (materials, components, logic structure), embedded knowledge (software), and interaction mechanisms (protocols, interface rules).

### Beyond the traditional view of firms: Innovative eco-systems

The nature of the firm as an organization is also affected by the pressures exerted by the new landscape. Ubiquitous computing and digitalization allow the convergence of many techno-scientific and techno-productive innovations, such as advanced digital manufacturing technologies (3D printing, advanced robotics, Internet of Things), new materials (bio- and nanomaterials, supermaterials), and new techno-scientific processes (data-driven production cycles, synthetic biology, post-genomics, data-driven scientific discovery, and applications of AI systems). The result is the dissemination of knowledge-intensive processes and outputs, where interdependencies, complementarities, cognitive and operational conflicts, and systemic integration become essential dynamic properties. In this scenario, it is not surprising that the boundaries between firms are not crisp anymore, but rather fuzzy and blurred, and they tend to be conceived in terms of innovative eco-systems.^44^ In fact, we posit that the study of companies’ decision making in the new landscape will miss the mark if it continues to focus on the firm as unit of analysis. The relevant unit of analysis is rather the bundle of organized processes that harness distributed information flows from the concert of sources we singled out in this paper. This means that the mechanism of formation of firms’ boundaries cannot be fully proxied by the classic make-or-buy trade-off or by simple transaction costs arguments. Firms, as micro-organisms in symbiotic (and in many cases parasitic, as the current energy and climate crisis has among its fundamental causes the excessive exploitation of the world’s stocks of natural assets) relation with the Earth system and immersed in the cyber-physical universe, are subject to continuous structural re-modulations, given the complex, ever-changing pressures and opportunities at multiple levels.

At the level of the economic and productive sequences, the variable sets of phases and operational tasks actors are involved in can now be modeled with computational tools in a systemic, multi-scale, and integrated perspective.^45^ This frequently includes the design of the structural properties of processes, the characteristics of outputs and performances, up to control along the entire life cycle of the products, whereas it is always possible to add functionalities, as we previously pointed out in subsection “new properties of processes and outputs.” All this takes place through top-down and bottom-up information-processing activities, in the context of deductive and inductive processes.

In summary, we witness the emergence of what Perez^2^ (see also Knell and Vannuccini^46^) would call a new techno-economic paradigm, which results from the combination of engineering, physical sciences based on computational modeling, and strategic management, strengthened by new and powerful information-processing technologies. In turn, these rely on multiple sources of data: structured (databases, spreadsheets), non-structured (written texts, photos, videos, images, and sound documents), and semi-structured (tags, markers useful for identifying certain elements, but for which it is not possible to develop models capable of giving them a structure). The importance of computational power and the ability to capture and store information from different sources cannot be underestimated; to that, we add a further essential factor: processing systems must have adaptive capacity, in the sense of not being limited to a static representation of the collected data and information. They must perform dynamic functions, in order to support the management of material and immaterial flows, as well as the creative interpretation of increasing information flows. To acquire adaptive capacity, actors must develop appropriate theoretical and applicative tools to capture information flows that go beyond consolidated cognitive frames; we will return to this in section “a paradigm shift for decision making.”

The ever-expanding cyber-physical universe incessantly generates sets of problems that scientists and experts from various disciplines strive to solve. A fundamental problem is that of making intelligible the growing mass of data and information, transforming them into useful knowledge to face perennially emerging economic needs. A logical implication of this is that there is always the need to overcome the gap between the computational capacity of agents (individual and collective) and the information generated by a constantly evolving environment. Precisely in relation to this gap, intrinsic to the decision-making processes of living beings, Herbert Simon developed the concept of bounded rationality.^47^ In situations characterized by limitations in computational power, the amount of time and memory, the approach proposed by Simon seems crucial to us, because the decision maker “is confronted with the problem of behaving approximately rationally, or *adaptively*, in a particular environment”^48^ (italics added). We consider this assimilation of the concept of approximately rational behavior to adaptive capacity particularly fruitful, whereby we will resume it in section “automation, augmentation, and the turing trap.”

Currently, the main trajectory pursued by agents to achieve adaptive capacity is to progressively augment decision-making processes with information-processing systems embedding AI. This is the building block of the cyber-physical universe we discuss next.

## Decision making in the new landscape

### Human decision making and AI

In the previous section, we introduced the idea that processing systems, in general and in particular within the cyber-physical universe, need to be dynamic and display adaptive capacity. This requirement brings us directly to the growing importance of the field of AI for decision making. In fact, an increasing strand of literature focuses on the possibility of strengthening human decision-making processes through the development of powerful software systems and their use in all activities at any level. Such software systems are, in essence, information-processing algorithms falling under the category of AI.

AI can be conceived as the last step in the development of a language for machines we illustrated in section “the trajectory (over centuries) toward the cyber-physical universe.” Over the decades, two fundamental approaches have been used in AI studies. The first is the classic paradigm of symbolic processing, or good-old-fashioned-AI (GOFAI), centered on the hypothesis of “physical symbol systems; that is, physical information-processing systems that process information based on “declarative knowledge bases.” In this case, the knowledge relating to the domain of a problem is represented through “declarative sentences” and it is processed through first-order logic. While classic AI analyzes well-defined problems using logic rules, the second approach is the sub-symbolic paradigm, explicitly inspired by the biological neural systems of the brain. Starting with the seminal book by Rumelhart and McLelland,^49^ neural computing, also known as connectionist approach, models processor-node networks without explicitly representing knowledge trough symbols. All (artificial) neural networks are directed graphs processing input into output having defined a certain activation functions for the nodes of the graph^24^; modern neural networks extend such topology to encompass multi-layered directed graphs and more modular and hierarchical structures (e.g., “capsules” as introduced by Sabour et al.^50^). The approach (from the initial experiments with perceptrons to current bio-inspired AI) tries to simulate *in silico* the individual and collective dynamics (rules of activation and propagation of information) of the neural networks that are activated in the brain.

After the first successes, the 1990s saw the latest among the cyclical “winters” of AI, because even the connectionist models (initially with only three layers of neurons) seemed to show limits in emulating cognitive functions such as language processing, perception, and memory. The consequence was loss of interest, reduction of investments in the research trajectory, and stasis in the creation of new, more sophisticated computational models. The connectionist approach gained new life in the early 2000s with the introduction of the deep learning technique.^51^ In short, deep learning applies the backpropagation algorithm based on gradient descent to update nodes weighting to a new organizational model of the artificial neural networks, made up of many layers (and thus deep), with groups of modules in each of them and transversal connections in an impressive numbers (billions). Deep neural networks (DNNs) models are showing remarkable performances in the recognition of spoken and written texts, images, and simple phonemes, reconstructing complex representations from simple and scattered typological details or categories. For example, a type of DNN, convolutional neural networks, uses the operation of convolution to extract feature from complex data input (e.g., images as grids of pixels), layering up these features from the most essential (corners, contours) to more articulated ones (full objects).

The success of deep learning in combination with artificial neural networks and the universe of new techniques and refinements developed in the last decade (for example, parallel advances in the technique of reinforcement learning) could not have been achieved without impressive advancements in computing power and in the availability of data.^24^ Increasing computational power and data availability are the by-product of the unfolding dynamics that lead to ubiquitous computing and connectivity: the generalized digitization of physical objects and processes that is at the core of the cyber-physical universe.

The last 20 years have witnessed an impetuous development of computerized systems and artificial agents capable of performing tasks and functions that normally require human intelligence. New methods and procedures with genetic algorithms turned out in the planning and control of optimization processes, while models based on neural networks have gradually assumed an increasingly important role in the recognition and processing of natural language and in artificial vision. Several scholars have developed Bayesian models of computational processing that combine structured knowledge representations with statistical inferential machines. Hierarchical Bayesian models have made it possible to discover “correct structural forms of many real-world domains,”^52^ as well as causal relationships and analogical transfers of knowledge in different domains. At the origin of these approaches are the contributions of Pearl,^53^ Muggleton and De Raedt,^54^ and Richardson and Domingos^55^: Pearl has developed Bayesian probabilistic models of causal relationships; Muggleton and De Raedt have contributed significantly to the inductive logic programming trend, which aims to create artificial systems capable of learning autonomously, through what is called statistical relational learning^56^; Richardson and Domingos introduced Markov logic networks, which consist of sets of formulas written in the logic of first-order predicates, with an assigned and variable weight based on experience and inductive processes. In an attempt to answer questions about how rich representations can emerge from partial, fuzzy, incomplete data, Lake and co-authors proposed the Bayesian Program Learning framework, based on three fundamental principles: compositionality, causality, and learning to learn.^57^ These are the founding elements of a process of construction based on cognitive blocks of inductive nature, which uses and reuses fragments of knowledge broken down and grouped in new forms according to probabilistic methods.

The evolution of the field of AI seems to follow a (tortuous) path toward model hybridization and to the definition of a unifying style that combines the properties of symbolic and sub-symbolic approaches.^58^ As every technology, and despite its potential, AI can be misused, especially given the “black box” nature of most algorithms, its brittleness and exposure to adversarial attacks, and its “garbage in, garbage out” essence, which makes AI systems very sensitive to the training data employed, data that very often reproduce patterns of bias present in the society.^59^ However, in terms of usefulness, AI technologies become a key tool to operate within the cyber-physical universe, given the need to incessantly process the exponentially growing (global) information flows. From our discussion, the particular form of interdependence between AI and the informational nature of the cyber-physical universe becomes clearer: AI is one of the participant technologies in the positive feedback cycle characterizing, to paraphrase Sraffa, the production of information by means of information (processing tools); that is, the circular relationship between advances in software systems (among which is AI) and the increase in computational power. Through this mechanism, information flows and the pervasive and ubiquitous digital world could grow exponentially and, in turn, became the new landscape on which to develop intelligence of socio-technical processes.

### Automation, augmentation, and the Turing trap

While we stress the importance of AI as a tool augmenting decision making, Brynjolfsson^60^ has highlighted the common fallacy assuming that most productivity-enhancing innovations (such as AI) relate to automation. This fallacy is reinforced by the evidence on the declining labor share (the fraction of total income allocated to wage/workers) in the economy, detected in almost all countries,^61^ with an accentuated trend in the United States.^62^ Many scholars identify the main cause of that in technical changes precisely because of an exclusive focus on automation, seen as “substitution-oriented automation” by technologists, businesspeople, and policy makers alike.^60^ These three categories are aligned in their vision of AI as a substitutive set of technologies: technologists are allured by the challenge of creating a human-like intelligence, fully recreating (rather than just emulating) *in silico* brain functions working autonomously; businesspeople are obfuscated by the imperatives of cutting costs and scale-up business models, so that they willingly interpret automation as replacement of labor; policy-makers are also captured by the narrative to automate rather than augment human knowledge and power. In this way, the feedback loop between the emergence of hyper-structures (section “coordinates for the current era: the cyber-physical universe”), automation instead of augmentation and socio-economic polarization produces concentration of technological and political power, suggesting an unattractive outlook: “the tendency of a greater concentration of technological and economic power to beget a greater concentration of political power risks trapping a powerless majority into an unhappy equilibrium: the Turing Trap.”^60^ The dilemma of automation versus augmentation is therefore posed, and this opposition contributions to redefine the foundations and the perspective with which to set up public and private decision-making processes.

## A paradigm shift for decision making

### Toward an adaptive strategic-thinking approach

Building on the analysis conducted so far, in this section we claim that, in order to be able to fit for the cyber-physical universe, decision making needs a proper paradigm shift. This shift must cover both general principles and operational criteria.

### General principles

The challenges connected with the cyber-physical universe require designing systems capable of withstanding temporary and structural shocks through the acquisition of resilience and robustness. Following Folke et al., we define resilience as “the capacity of a system to absorb disturbance and reorganize while undergoing change so as to retain essentially the same function, structure, identity, and feedbacks.”^63^ Instead, the robustness of a system indicates the decisional and structural flexibility suitable for absorbing in the long-term changes induced by fluctuating environments.^64^ Studies on this subject show that two general principles favor both properties: redundancy and modularity. Redundancy (“the property of one component to perform another’s function”^64^) can avoid catastrophic effects resulting from the loss of specific components, such as to generate cascading effects in the event of systemic interdependencies. Modularity means “compartmentalization, or the decomposition of a system into discrete units, into subsets of entities with high-frequency interactions between them and low-frequency interactions between subsets.”^65^ Modularity confers strength, because it reduces the possibility of spreading negative impulses, similarly to the social distancing the world has been experiencing during the COVID-19 pandemics.

Uncertainty and complexity are immanent in decision-making processes in the novel global landscape. To understand that in more depth, we resort to the framework proposed by Courtney et al.,^66^ who outline four levels of uncertainty.

Level 1 uncertainty occurs when uncertain elements are of no particular relevance to decision making, because they pertain, so to speak, to non-high-level operational contexts. A concrete example could be the purchase of a particular type of software in a defined range of substantially equivalent products. The aura of uncertainty here concerns the evaluation of which is the most appropriate alternative for the actors’ specific reality.

Level 2 uncertainty is found when possible alternatives are known to the point of being able to assign probabilities to each of them. In these cases, multiple scenarios can be outlined, for which a reliable estimate of the trend impact in technological, economic, and social terms can be made. An example of such contexts is the decision of integrated circuit manufacturers regarding the choice of semiconductor material for their devices (e.g., silicon versus graphene) given their different properties and trade-offs in use for production.

Level 3 uncertainty identifies situations in which a set of potential technological trends can be outlined, or related to consumption and production models, but not in a way to be precisely defined and in any case based on a set incomplete and fuzzy knowledge. For instance, currently the trend toward “Industry 4.0” is reliably defined in broad lines; however, the modalities of implementation at the company, sector, and territorial level, as well as the possible implications in terms of work organization, skills, and organizational model of the techno-productive sequences, are not clear. Under level 3 uncertainty, we can include a large part of today’s decision-making processes in the technical-scientific, economic-productive, and health fields.

Finally, level 4 uncertainty is encountered if the interactions between the fundamental dimensions needed to take a decision and uncertainty are such as to create an environment that is virtually impossible to predict. A current example is the evolution of quantum computation.^67^

The cyber-physical universe frequently generates situations with uncertainty levels 3 and 4. In this context, an approximately rational, or adaptive, approach^48^ becomes essential for decision makers, because a merely responsive behavior toward unknown events can be self-destructive. Decision makers must adopt an open mindset and incessantly explore new knowledge domains, through the adoption of flexible and agile cognitive frames, without giving up abruptly to their existing knowledge base. To this end, an adaptive approach à la Simon can be pursued through a mix of exploitation and exploration activities.^68^ In summary, in the new landscape, new strategic imperatives emerge for decision makers: continuous scanning of the technical-scientific frontier, continuous updating of knowledge, and continuous rethinking of strategies and behaviors.

### Operational criteria

From the strategic principles just discussed derive no less important operational criteria to be followed in a scenario characterized by acceleration, uncertainty, and unpredictability. First, the fragility of the “command-and-control” paradigm—the pursuit of maximum efficiency according to mental and organizational schemes planned in conditions of complete knowledge of the operating environment—emerges clearly. As a complete insulation from the complexity of the cyber-physical universe is impossible, redundancy, considered inefficient in a controlled environment, become essential to guarantee operating conditions when more or less sudden shocks arise, as has been the case with global supply chain disruptions of 2020–2022.

Connected to this, we identify a second criterion: the time horizon of each agent must extend beyond short-term expectations; rather, it must be long-term oriented and incorporate a focus on interdependencies and on multi-dimensional feedback loops within the whole Earth system. In a hyper-connected world, actors must be patient, as interdependencies make contexts highly variable and less predictable; learning processes become crucial and the application of “mechanical management” models based on bounded sets of choices to maximize loses value, because the variability of the parameters that are useful for decisions makes optimization only a transient result. Thus, adaptive strategic thinking and adaptability become fundamental, as they are based on incessant research activity in three directions^69^: (1) exploration of the technical-scientific potential, (2) analysis of systemic interrelationships and multiple risks, and (3) transformation of operational models according to the identified trajectories.

### How to face the present challenges, reversing the prevailing pattern of techno-scientific advancements

As a final building block of our analysis, let us summarize some of the main problems and challenges related to the emergence of the cyber-physical universe, which are also guidelines for the research activity necessary to face the challenges looming at the horizon for humanity.

First, the opposition between automation (conceived as a replacement) and augmentation of labor is a predominant feature of the current technical-economic evolution. Second, the new technologies at the core of the cyber-physical universe foster a misalignment of incentives, thereby fueling a growing concentration of power and wealth. Technologies and tools invented by humanity during its evolution are the result of what the French anthropologist Leroi-Gourhan^70^ called the “imaginary freedom” of humans, who shaped them and are shaped by them. Human beings “reengineered the environment and themselves […] Tools become part of the environment that shapes our beliefs, preferences, and capabilities.”^71^ What we have labeled the cyber-physical universe derives from the “transformative power” of modern information technologies and is the fulfilment of a long-term journey of human evolution along the steps we presented in section “the trajectory (over centuries) toward the cyber-physical universe”: “… especially since the invention of writing, a dematerialized image has been formed – an image essential to mental development and to progress, but one which, in the human sciences especially, has led to the denial of any connection between the human and the rest of the living world.”^70^ We have created powerful tools, which shape us in ways we did not foresee: “[I]nstrumental reason, triumphant technique, and unbridled science are addictive. They create a concrete reality, a self-fulfilling nightmare.”^72^ In other terms, in the new landscape there are increased risks of an extreme amplification of McLuhan’s message: “‘The medium is the message’ because it is the medium that shapes and controls the scale and form of human association and action.”^73^ While, in the past, the formation and evolutionary process of personal identity took place fundamentally on the basis of physical-visual interactions, now information technologies and their networked nature play an increasingly important or even preponderant function in “mediating” interactions between people. They become the tools through which perceptions are modeled, cognitive processes evolve, and therefore humans act on the operating environment, which in turn acts on us. Ultimately, personal identities are formed on the basis of belonging, directly or indirectly, to global networks. We have become “networked inhabitants of the emerging information society.”^74^ In the cyber-physical universe we live in, humans think and act in algorithmic social systems, where self-selective mechanisms, cognitive asymmetries, information polarization, and concentration of power and wealth are induced by self-reinforcing pressures.

In a sense, the notion of cyber-physical universe relates to Wiener’s ideas on “the human use of human beings,”^75^ as it might act as a grand machinery to reduce humans to the status of “cods and levers and rods.” Furthermore, with our analysis we confirm that today’s socio-economic systems are characterized by alignment problems of a dual nature: direct and social. We take this distinction from Korinek and Balwit,^76^ extending and changing its scope. They defines the two terms in reference to AI, while we believe it is useful to expand the field of application to information technologies in general. Direct alignment refers to whether new frontier technologies are pursuing goals that are consistent with the goals of their operators, defined as “the entit[ies] that [are] creating, operating, and controlling [the new technologies]”^76^ (our changes are in square brackets). Social alignment refers “to whether [the new technologies are] pursuing goals that are consistent with the broader goals of society, taking into account everybody who is affected by the system and internalizing any externalities.”^76^

The problems identified in this section, together with the three global joint crises we presented at the outset of the paper, indicate that misalignment prevails in an extremely wide range of individual and collective decision-making processes, at multiple scales: from local communities to the global scale. The general consequence is that the entire planet as a techno-social systems (section “coordinates for the current era: the cyber-physical universe”) is at risk. Therefore, the insight to be derived from our analysis is that a radical change is required in order to reverse the main components of the highlighted techno-scientific and socio-technical evolutionary patterns. Such radical change requires the overcoming of models anchored to a fixed set of tools and the adoption of models grounded on the continuous search for new tools. In the context just described, the indication of the transition from mechanical management, based on the search for permanent and definitive solutions to complex problems, to “biological thinking,”^77^ centered on very different principles, is suggestive: experimentation, resilience instead of efficiency, systemic and holistic vision, plurality of choices, an tools and skills to develop adaptive potentials become key vectors for strategy.

Ultimately, it seems to us that adaptive strategic thinking is the theoretical and operational perspective suitable for making the most of the potential that is opening up to humanity, even if unknowns and risks are looming. This might be the key approach to allow humanity to be able to maintain control of the “fabric of reality,” which otherwise can be subject to uncontrolled dynamics with devastating effects. It is difficult to hypothesize that a multi-dimensional, complex, and systemic vision can emerge without a conscious processing, both individually and collectively; hence, this paradigm shift is essential to gain awareness of the multi-scale and global nature of the changes taking place, and therefore to develop fit strategies for survival.

## Conclusions

In this paper, we uncovered the core principles, dynamics, drivers, and patterns behind the emergence of what we called the cyber-physical universe. The cyber-physical universe is the fundamentally novel landscape in which agents operate, molded by the ubiquitous nature of information and the generative dynamics its production and use entails. The information technologies that are the infrastructure of the cyber-physical universe become the fabric of reality that blends together physical and virtual (digital) processes.

Through continuous approximations, we focused first on the emergence of the cyber-physical universe as the common playground or workspace in which processes of different nature take place. Then, we singled out the historical steps that drove humanity toward the cyber-physical universe, and described some identifiable patterns occurring in the current landscape. From there, we unbundled the complexity that characterizes the intertwined forces at work in this context in order to shed light on which opportunities and challenges actors face in the novel ontological space. In particular, we described transformations in the mode of conducting production activities, in the boundaries of the firm, and in decision-making processes. The latter, in particular, is subject to a proper paradigm shift, due to advances in AI that can help and augment decision making. The dynamic matching between progress in autonomous decision making system and restless mutations in the search-problem-solution space—both by-products of the rapid evolution of the cyber-physical universe—requires the adoption of principles and operational criteria that are less inspired by the command-and-control approach and more fit to capture the non-linearities of the novel landscape. This is particularly relevant given the challenges that the cyber-physical universe casts on humanity, in particular problems of alignment between the space of potentialities that come with the new fabric of reality and the impact that these have on actors’ incentives, interactions, and mental models. We suggested that a frame based on adaptive strategic thinking would be the most appropriate for actors to continue thriving in the current era of deep and extended changes.
